# Motor and Cerebellar Architectural Abnormalities during the Early Progression of Ataxia in a Mouse Model of SCA1 and How Early Prevention Leads to a Better Outcome Later in Life

**DOI:** 10.3389/fncel.2017.00292

**Published:** 2017-09-20

**Authors:** Mohamed F. Ibrahim, Emmet M. Power, Kay Potapov, Ruth M. Empson

**Affiliations:** Department of Physiology, School of Biomedical Sciences, Brain Health Research Centre, Brain Research New Zealand, University of Otago Dunedin, New Zealand

**Keywords:** cerebellum, ataxias, climbing fibers, Purkinje cells, mouse models

## Abstract

Exposing developing cerebellar Purkinje neurons (PNs) to mutant Ataxin1 (ATXN1) in 82Q spinocerebellar ataxia type 1 (SCA1) mice disrupts motor behavior and cerebellar climbing fiber (CF) architecture from as early as 4 weeks of age. In contrast, if mutant ATXN1 expression is silenced until after cerebellar development is complete, then its impact on motor behavior and cerebellar architecture is greatly reduced. Under these conditions even 6 month old SCA1 mice exhibit largely intact motor behavior and molecular layer (ML) and CF architecture but show a modest reduction in PN soma area as a first sign of cerebellar disruption. Our results contrast the sensitivity of the developing cerebellum and remarkable resilience of the adult cerebellum to mutant ATXN1 and imply that SCA1 in this mouse model is both a developmental and neurodegenerative disorder.

## Introduction

Spinocerebellar ataxia type 1 (SCA1) is a degenerative and progressive autosomal dominant disorder caused by expansion of a CAG repeat in the gene for the transcriptional regulator Ataxin-1 (ATXN1; Orr et al., 1993). SCA1 is one of up to 40 different types of human SCAs. In human SCA1, patients show loss of motor coordination and posture in their early forties that progress over the next 10–20 years (Wagner et al., 2016). In a recent study of a subgroup of SCAs, subjects diagnosed with SCA1 in their late thirties were the fastest to progress and the number of CAG repeats related directly to the severity of the symptoms (Jacobi et al., 2015). Despite the appearance of the disorder in later life the CAG expansion in the ATXN1 gene is present throughout life but might exert its most damaging influence amidst critical developmental changes in cerebellar gene expression.

ATXN1 normally interacts with a number of transcriptional regulators via its AXH domain and both gain and partial loss (Lim et al., 2008) of this function leads to a multitude of gene network expression changes in SCA1 (Ingram et al., 2016). The interaction between ATXN1 and the transcriptional repressor Capicua (Lam et al., 2006) and loss of the developmental transcription factor RORalpha (Serra et al., 2006) both directly contribute to cerebellar pathology and the progression of SCA1. Disrupted RORalpha dependent gene expression networks in a mouse model of SCA1 are particularly relevant to previously suggested links between altered cerebellar development and the ataxias (Manto and Jissendi, 2012). Specifically, RORalpha mediates changes in the expression of calcium handling proteins and metabotropic glutamate receptors (mGluR1; Serra et al., 2004, 2006). Both functions are critical during the first few postnatal weeks when controlled expansion of the Purkinje neuron (PN) dendrite in the molecular layer (ML) and climbing fiber (CF) inputs are refined (Kano et al., 1995; Boukhtouche et al., 2006; Dusart and Flamant, 2012). Notably, shrinkage of the ML and the PN dendrites with retraction of CFs are architectural hallmarks of SCA1 (Duvick et al., 2010; Barnes et al., 2011; Ebner et al., 2013; Power et al., 2016) and other ataxias, for example those where calcium signaling proteins (Matsumoto et al., 1996; Empson et al., 2007) and mGluR1 are knocked out (Kano et al., 1997; Ichise et al., 2000).

Using a mouse model of SCA1 where mutant ATXN1 expression can be silenced, or switched off, using TET-OFF engineering (Zu et al., 2004) we ask if mutant ATXN1 exposure during the critical period of cerebellar development is more damaging than mutant ATXN1 exposure in adulthood, and if so, what are the earliest hallmarks of the beginnings of the disease. Previous work used the TET-OFF mouse model to show that silencing mutant ATXN1 expression after disease onset ameliorated the disease at its later stages (Zu et al., 2004) and that full reversal occurs if silencing happens early in the disease. In a separate study (Serra et al., 2006) silencing ATXN1 in the first 2 or 3 weeks of developmental life, and then allowing mutant ATXN1 expression for 12 weeks ameliorated ML shrinkage but the behavioral consequences or the extent of rescue and other architectural changes were not examined. Here, we assessed motor performance and cerebellar architecture early in the development of the SCA1 model, from 4 to 12 weeks of age. Then, in separate groups of these mice we delayed mutant ATXN1 expression (as in Serra et al., 2006) but for a period of 6 weeks when development of PN physiology is complete (Arancillo et al., 2015), and monitored their motor behavior and cerebellar architecture for up to 6 months alongside age-matched controls.

We found that exposure to mutant ATXN1 in young, developing mice caused ataxia and disrupted CF, but not ML, architecture from as early as 4 weeks of age; in contrast mice exposed to ATXN1 from 6 weeks of age when cerebellar development was over, exhibited normal behavior and cerebellar architecture remained largely intact for up to 6 months. Our results highlight SCA1 in mice as both a developmental and neurodegenerative disorder.

## Materials and Methods

### Transgenic Mice

We used homozygous male and female SCA1 82Q Tre/Tre; tTA/tTA (82Q mice) expressing mutant human ATXN1 with a pathological 82Q (CAG) repeat specifically within the cerebellar PNs in a TET-regulated manner. These mice were generated by crossing mice expressing tTA specifically in PNs under the control of the Pcp2/L7 regulatory region and mice expressing mutant ATXN1 under the TRE control element (Zu et al., 2004). We used FVB/pcp2 tTA/tTA (WT mice) as our control mice, founder mice were kindly provided by Orr et al. (1993) (University of Minnesota, Minneapolis, MN, USA).

### Conditional Mutant ATXN1 Expression

To understand the effects of delaying mutant ATXN1 expression in the developing cerebellum, we turned off the mutant ATXN1 gene for the first 6 weeks of age (6 OFF) by giving 200 mg/kg doxycycline (dox) in the diet of pregnant mothers and their litters. In this mouse dox treatment represses mutant *Atxn1* mRNA and ATXN1 protein expression after 24 h and 2 days respectively (Zu et al., 2004). After 6 weeks we removed dox from the diet when mutant ATXN1 expression should be fully restored within a week (Serra et al., 2006; Barnes et al., 2011) and then followed the mice to 6 months of age.

All animals were housed in groups in a temperature (21 ± 2°C) and humidity (50% ± 20%) controlled room with a 12 h day/night cycle. Animal care and all procedures were carried out according to University of Otago guidelines that align with national and international standards and ethical approval.

### Motor Performance

Both male and female groups of mice were used and tested separately in a purpose built sodium lighting room on the same time each day. Motor performance was assessed using an accelerating rotarod (Rotamex 5, Columbus Instruments, Columbus, OH, USA), for four consecutive days and on each day mice were tested for four trials, with a 10 min interval between each trial. During each trial the rod accelerated from 4 to 40 RPM over a 5 min period. When the animal fell from the rod or completed a passive rotation by clinging on to the rod this was considered to be the end of the trial, and the trial time to fall from the rod (or latency to fall) was recorded. We calculated the mean value from each of the four trials to establish the latency to fall for each mouse on each day.

### Immunohistochemistry

Three-hundred micrometer thick sagittal cerebellar vermis slices were cut in cold artificial CSF (aCSF) containing the following (in mM): 126 NaCl, 3 KCl, 1 NaH_2_PO_4_, 26 NaHCO_3_, 2.4 CaCl_2_, MgCl_2_, and 10 glucose and then were fixed in 4% paraformaldehyde in PBS. After washing in PBS and 0.3% TRITON X-100 in PBS, slices were incubated overnight in primary antibodies against guinea pig calbindin to measure ML height and against rabbit vesicular glutamate transporter (vGlut2) to measure CF extension (guinea-pig, 214005; rabbit, 135405 respectively, both Synaptic Systems; 1:500 concentration). After incubation, the slices were washed three times in PBS and were incubated with secondary antibodies for 4 h at room temperature (AlexaFluor 647 and 555-conjugated antibodies both Life Technologies, 1:500 concentration). Images were obtained using a Nikon A1R confocal microscope (1.24 μm/pixel x, y, 561 and 638 nm laser excitation).

### Quantification of ML Height, CF Extension and PN Soma Area

We measured ML height and CF extension from folia III, IV or V. ML height is calculated as the distance from the base of the PN to the top of the dendritic tree using the calbindin labeling as our guide (Zu et al., 2004; Power et al., 2016).

We used two alternative methods to assess CF morphology, both based upon measurement of the extension of the vGlut2 labeled CFs relative to the ML height.

First, we expressed CF extension as a ratio of CF (vGlut2)/ML (calbindin) height as previously (Barnes et al., 2011; Power et al., 2016). Briefly, we used a 634 μm × 634 μm field of view from simultaneously collected fluorescence-based confocal images for calbindin and vGlut2 labeling from two adjacent folia (III, IV or V) using a line to determine the extent (length) of calbindin labeling to determine the ML height, using ImageJ (Schneider et al., 2012). We then switched to the vGlut2 labeled image and used the same line to identify the (always shorter) extent of the nearest set of CFs. We made six measurements of this type from each slice at the middle, left and right sides of the field of view. The mean value from all six measurements provided single ML height and the CF extension values for one slice. From two slices per animal we generated a single mean value for each animal. In separate experiments (*n* = 3 mice) we found no significant differences between ML height or CF extension (CF(vGlut2)/ML(calbindin) ratio) across folias III–V; for ML *F*_(2,6)_ = 0.66, *P* = 0.55; for CF extension *F*_(2,6)_ = 1.43, *P* = 0.31, one way ANOVA, data not shown.

Second, we took inspiration from Kuo et al. (2017) to make measurements of CFs that extend beyond 70% of the ML height. Briefly, with Image J, using the same fields of view as above, we placed a 200 μm wide rectangle box over the full extent of the ML, orienting the ML appropriately. We adjusted the rectangle size to 70% of its height and used the vGlut2 labeled image to count the number of CF (vGlut2 labeled structures) that extended beyond the height of the rectangle. We measured CF number in this way from two different folia and from two slices, creating a single mean value for each mouse. Two independent assessors generated similar values from the same fields of view across three separate datasets.

To measure PN soma area we outlined the soma using the free-hand selection tool of ImageJ (Schneider et al., 2012) not including the apical dendrite, and calculated the area. We analyzed between 400–800 PN soma from at least three slices from a single animal. Since the distribution of the soma sizes was not normal (*P* < 0.05, Shapiro-Wilk normality test) we used the median values to represent the value for each animal, the distribution of median soma size from each animal group were normally distributed.

All data were tested for normality (Shapiro-Wilk normality test) and where normal (*P* > 0.05), the values were expressed as the mean ± standard error of the mean (SEM). For comparisons we used two-way repeated-measures ANOVA using values from WT and treated mice (column effects) to compare motor behavior, or one-way ANOVA using values from WT and treated mice (between columns) to compare ML height and CF retraction, alongside Bonferroni’s multiple comparisons in Graphpad Prism 7.

## Results

### SCA1 Mice Exhibit Early Deficits in Motor Behavior Whereas Mice Exposed to Mutant ATXN1 after Cerebellar Development Exhibit Wild Type Levels of Motor Behavior

**Figure 1:**
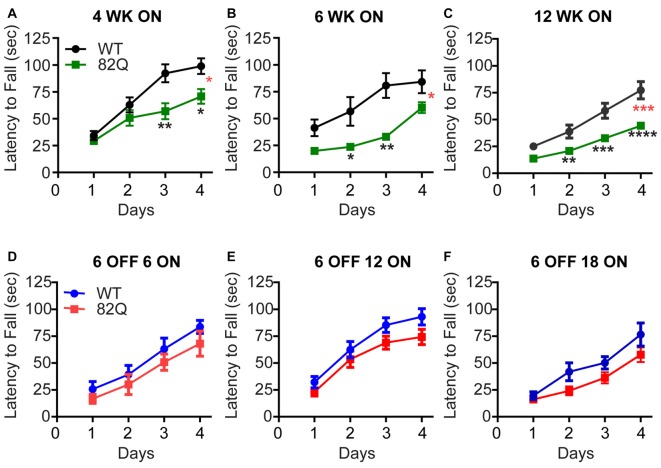
Reduced rotarod performance of spinocerebellar ataxia type 1 (SCA1) 82Q mice from as early as 4 weeks of age that is rescued in mice up to 6 months of age by silencing the ATXN1 gene during the first 6 weeks of life. **(A–C)** Reduced motor performance on the accelerating rotarod (latency to fall from the rod) by **(A)** 4 week old (4 WK ON), **(B)** 6 (6 WK ON), and **(C)** 12 (12 WK ON) ATXN1 overexpressing SCA1 82Q mice compared with age matched wild type (WT) mice. Note the reduced ability of all ages of 82Q SCA1 mice to learn the motor task over subsequent days, and the reduction in performance as the WT mice age. Four weeks *N* = 15 WT and *N* = 14 82Q, *F*_(1,28)_ = 7.1, *P* = 0.0126, represented by (*, red); 6 weeks, *N* = 8 WT and *N* = 7 82Q, *F*_(1,13)_ = 8.0, *P* = 0.0142, represented by (*, red) 12 weeks, *N* = 17 WT and *N* = 20 82Q, *F*_(1,35)_ = 17.6, *P* = 0.0002, represented by (***, red) all from the two-way ANOVA; * represents *P* < 0.05, ** represents *P* < 0.01, *** represents *P* < 0.001, **** represents *P* < 0.0001 in *post hoc* Bonferroni comparisons within the two way ANOVA. **(D–F)** Turning off expression of mutant ATXN1 for the first 6 weeks of life (6 OFF) with doxycycline and then turning the expression ON by removing doxycycline from the diet, preserved motor performance of SCA1 82Q mice for an additional 18 weeks until the mice reached 24 weeks (6 months) of age, **(D)** 12 weeks (6 OFF 6 ON), **(E)** 18 weeks (6 OFF 12 ON), and **(F)** 24 weeks (6 OFF 18 ON); *F*_(1,14)_ = 1.2, *P* = 0.29 6 OFF 6 ON *N* = 6 82Q *N* = 10 WT mice; *F*_(1,36)_ = 2.9, *P* = 0.1 6 OFF 12 ON *N* = 17 82Q *N* = 21 WT mice; *F*_(1,29)_ = 3.5, *P* = 0.07 6 OFF 18 ON *N* = 17 82Q *N* = 14 WT mice, all from two-way ANOVA. Symbols represent mean values and error bars are standard error of the mean (SEM).

SCA1 82Q mice show deficits in their motor performance on the accelerating rotarod as early as 4 weeks of age (Figure 1A) when we observed a reduced capacity of the mice to improve on subsequent daily trails compared with age-matched and WT control mice. Two weeks later we observed further impact of the continued mutant ATXN1 expression by a reduced initial performance of the mice on Day one, compared with WT that was also accompanied by slower learning by the mice on the subsequent daily trials (Figure 1B). At 12 weeks of age the SCA1 82Q mice exhibit further reduced capacity to improve their performance on the rotarod, consistent with the previously reported progression of the disease from early to mid-stage (Burright et al., 1995; Zu et al., 2004). In contrast, SCA1 mice that were exposed to mutant ATXN1 only after 6 weeks (using dox-induced repression of 82Q in this mouse (Zu et al., 2004)) motor behavior was largely intact even after 18 weeks of exposure to mutant ATXN1 in 6 month old mice (Figures 1D–F), 6 OFF 18 ON. These mice exhibited slightly (but not significantly) reduced learning during the daily trials, compared with dox-treated age matched WT controls (*P* = 0.07) but the mice exhibit a similar Day one performance as the WT controls. This pattern is reminiscent of the deficit we observed at the early, 4 week, stage of the disease (Figure 1A). These results highlight how delaying expression of mutant ATXN1 until after 6 weeks of age when anatomical and physiological development of PNs is complete (Kaneko et al., 2011; Arancillo et al., 2015) very significantly prevents the progression of the disease and allows the mice to perform at wild type levels for many months.

### Cerebellar ML Architecture Is Disrupted in the Very Early Stages of SCA1 but Not in Mice Exposed to Mutant ATXN1 after Cerebellar Development

Given the reduction of motor performance at 4 weeks of age and the largely intact motor performance of the SCA1 mice receiving delayed ATXN1 expression, we next sought to assess the cerebellar architecture in these mice. We detected the calcium binding protein calbindin (Cb), heavily expressed by PNs (Bastianelli and Pochet, 1993), to examine the ML height and PN dendrite expansion as well as the soma size. We also identified CF terminals in the ML, as measured by their specific expression of the pre-synaptic excitatory marker, the vGlut2 (Fremeau et al., 2001). We measured CF architecture using two methods; firstly the extent to which the terminals reach into the ML, normalized to the ML height (Barnes et al., 2011) and secondly the number of CFs that reach beyond 70% of the ML height (Kuo et al., 2017).

**Figure 2:**
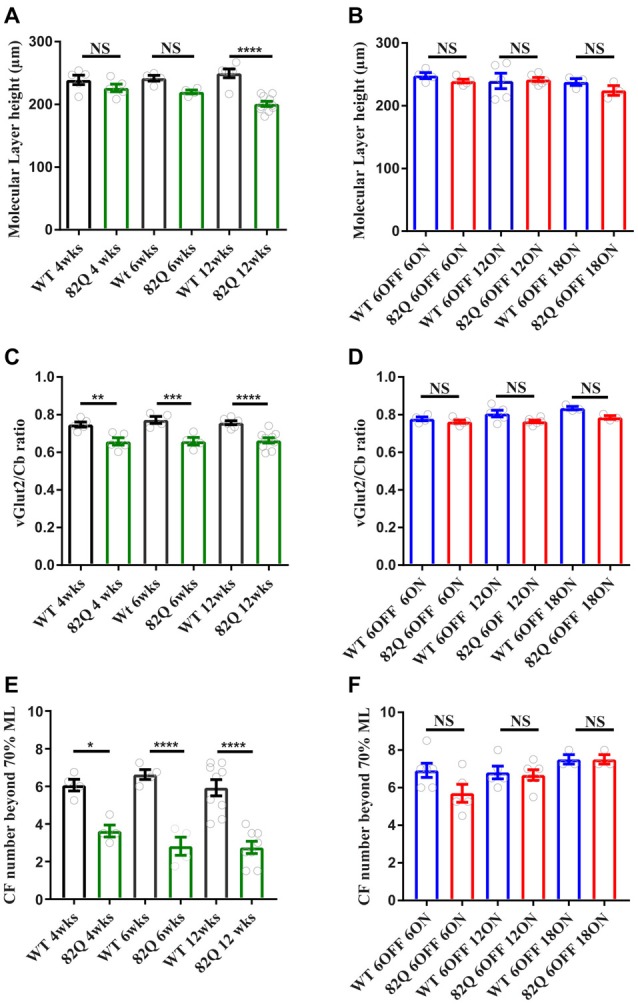
Altered cerebellar molecular layer (ML) and climbing fiber (CF) architecture in SCA1 82Q mice from as early as 4 weeks (wks) of age that is rescued in mice up to 6 months of age by silencing the ATXN1 gene in the first 6 weeks of life. **(A)** Intact ML height in 4 and 6 week old 82Q mice that shrinks in 12 week old 82Q mice as the disease progresses. NS and **** represent *P* > 0.05 and *P* < 0.0001, respectively in one-way ANOVA Bonferroni multiple comparisons; *N* = 5 WT and SCA1 82Q 4 week old mice; *N* = 4 WT and SCA1 82Q 6 week old mice, *N* = 6 WT and *N* = 10 SCA1 12 week old mice.** (B)** ML shrinkage is prevented (NS, *P* > 0.05 one-way ANOVA *post hoc* Bonferroni comparisons) in 12 week (6 OFF 6 ON), 18 (6 OFF 12 ON) and 24 week (6 OFF 18 ON) old SCA1 82Q mice; *N* = 5 WT *N* = 6 SCA1 82Q mice 6 OFF 6 ON; *N* = 5 WT, *N* = 6 SCA1 82Q 6 OFF 12 ON mice, *N* = 3 WT and *N* = 3 SCA1 82Q 6 OFF 18 ON mice. **(C)** Retracted CF innervation (labeled with vesicular glutamate transporter (vGlut2)) relative to the ML height (Cb) **P < 0.01, ***P < 0.001 and ****P < 0.0001 in one-way ANOVA Bonferroni multiple comparisons, and **(D)** reduced number of CF (vGlut2 structures) extending beyond 70% of the ML height (Cb) in 4, 6 and 12 week old SCA1 82Q mice, *P < 0.05, ****P < 0.0001 in one-way ANOVA Bonferroni multiple comparisons, *N* values as in **(A,B,D–F)** show rescue of cerebellar architecture in dox treated SCA1 82Q mice compared with age-matched dox treated WT mice, at 12 (6 OFF 6 ON), 18 (6 OFF 12 ON) and 24 weeks of age (6 OFF 18 ON). NS represents *P* > 0.05 one-way ANOVA Bonferroni multiple comparisons. Symbols represent mean values from each animal, bars are mean values and error bars represent SEM.

**Figure 3:**
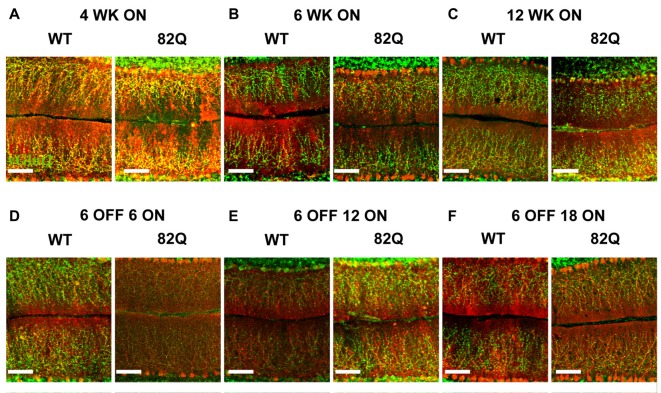
Altered cerebellar ML and CF architecture in SCA1 82Q mice from as early as 4 weeks of age that is rescued in mice up to 6 months old by delaying mutant ATXN1 gene expression until after the first 6 weeks of life. Example images of calbindin (red) and vGlut2 (green) immunofluorescence showing ML height and CF respectively in **(A)** 4 week (WK), **(B)** 6 week and **(C)** 12 week old 82Q mice, CF is retracted in all three age groups to a similar extent (see also Figure 2C). Note the shrunken ML in 12 week old SCA1 82Q mice **(C)**. Note intact ML and CF architecture in SCA1 82Q mice of all ages where ATXN1 expression was silenced (turned off) in the first 6 weeks of life, **(D)** 12 weeks (6 OFF 6 ON), **(E)** 18 weeks (6 OFF 12 ON), and **(F)** 24 weeks (6 OFF 18 ON) old WT and SCA1 82Q mice. Scale bars 100 μm.

In 4 week old SCA1 mice that displayed motor behavior deficits, we observed a significant reduction in both CF measurements indicating a retraction of the CF down the PN dendrite (Figures 2C,E, 3A) with no associated reduction in the ML height (Figures 2A, 3A). We observed the same pattern in 6 week old SCA1 82Q mice (Figures 2A,C, 3B) but by 12 weeks of exposure to mutant ATXN1 the ML was clearly reduced and both CF measurements indicated clear disruption of CF innervation (Figures 2A,C,E, 3C). These results indicate that significant CF retraction is an early indicator of disrupted cerebellar architecture in 4 week old SCA1 mice accompanying the impaired motor behavior (Figure 1A), and that ML shrinkage is likely a secondary phenomenon as the disease progresses.

In contrast, the cerebellar architecture (ML height and CF retraction, Figures 2B–F, 3D–F) from mice exposed to mutant ATXN1 after development, remained similar to age-matched doxy-treated WT controls, consistent with their largely intact behavioral performance (Figures 1D–F).

### PN Soma Area is Reduced in 12 Week Old SCA1 82Q Mice and Also Following 18 Weeks of Exposure to Mutant ATXN1 after Cerebellar Development

**Figure 4:**
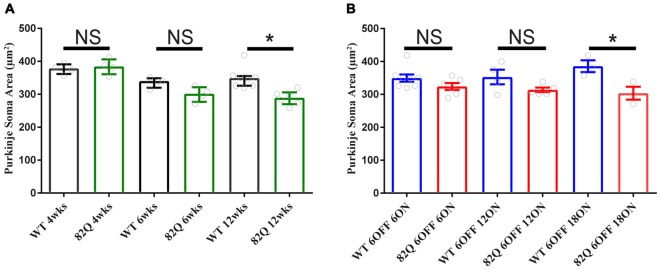
Purkinje neuron (PN) soma shrinkage in 12 week old ataxic SCA1 82Q mice and in pre-symptomatic 6 month old (6 OFF 18 ON) SCA1 82Q mice. **(A)** PNs soma area is normal in 4 week (wks) and 6 week old SCA1 82Q mice compared with WT age matched controls, but shrunken in 12 week old SCA1 82Q mice. **(B)** PN soma area is preserved in SCA1 82Q 6OFF 6ON, 82Q 6OFF 12ON, and mildly shrunken in SCA1 82Q 6OFF 18ON mice. One-way ANOVA, Bonferroni multiple comparisons, 4 weeks *N* = 4 WT and SCA1 82Q, 6 weeks *N* = 4 WT and SCA1 82Q, 12 weeks *N* = 8 WT and 6 SCA1 82Q; 6 OFF 6ON, *N* = 8 WT and *N* = 6 SCA1 82Q, 6 OFF 12 ON *N* = 4 WT *N* = 5 SCA1 82Q, 6OFF 18ON *N* = 3 WT AND *N* = 3 SCA1 82Q. *P < 0.05. Bars represent mean of median soma areas (symbols represent median values from each animal) and error bars represent SEM.

Mutant ATXN1 subverts PN nuclear transcriptional pathways at the soma (Skinner et al., 2002), so we also examined PN soma size to determine if this parameter is a useful marker of SCA1 progression. We found that PN soma area was reduced in 12 week old SCA1 mice perhaps reflecting the overall shrinkage of the cell as the PN dendrites are clearly shrunken at this age. Indeed, soma size was not significantly changed in PNs from 4 or 6 week old SCA1 82Q mice when the ML was also not significantly reduced (Figure 2A). In mice exposed to mutant ATXN1 only after development, the PN soma area remained similar in doxy-treated and age-matched control mice except after 18 weeks of mutant ATXN1 exposure when we observed a modest but significant reduction in PN soma area (Figure 4B), even though the ML was not shrunken.

## Discussion

Exposing developing cerebellar PNs to mutant ATXN1 disrupts motor behavior and cerebellar CF architecture prior to ML shrinkage in 82Q SCA1 mice from as early as 4 weeks of age. In contrast, if mutant ATXN1 expression is turned on after cerebellar (and CF) development is complete, even 6 month old adult SCA1 mice exhibit near-normal motor behavior and cerebellar architecture. We find it remarkable that long term prevention of mouse SCA1 is possible in this way and our results build upon the earlier findings from Serra et al. (2006). That early prevention is so effective, and for so long, contrasts with the partial recovery of mid-stage mouse SCA1 when mutant ATXN1 expression is reversed during disease progression (Zu et al., 2004). The implications are that mutant ATXN1 is far more damaging to developing cerebellar circuits than to the adult cerebellum and that SCA1 in this mouse model is both a developmental and a neurodegenerative disorder. Whether this is the case in humans is hard to predict especially as clinically, SCA1 is regarded as a relatively late onset disease.

In mice, cerebellar PNs develop rapidly during the first three postnatal weeks (Goldowitz and Hamre, 1998) when a critically coordinated sequence of gene expression takes place to establish PN and cerebellar ML architecture (Leto et al., 2016). ATXN1 interacts with a variety of transcription factors to influence gene expression profiles and mutant ATXN1 disrupts gene expression profiles as an underlying cause of SCA1 (Serra et al., 2004, 2006; Lam et al., 2006; Ingram et al., 2016). This disruption during the critical period of cerebellar development seems to be particularly damaging as even 4 week old SCA1 mice show motor deficits (Figure 1A). Just two more weeks of mutant ATXN1 expression further accelerates the motor dysfunction to the level of 12 week old SCA1 mice (Figures 1B,C). However, if exposure to mutant ATXN1 is delayed until after the critical period of cerebellar development (6 weeks after birth), motor performance (similar to age-matched doxy-treated wild type mice) remains intact even after 18 weeks of exposure to mutant ATXN1 in 6 month old mice (6 OFF-18 ON, Figures 1E,F). This result highlights the remarkable resilience of the adult cerebellum and is consistent with the relative sparing of the cerebellum in adult onset neurodegenerative disorders such as Alzheimer’s where the cerebellum is often used as a reference during human functional imaging (Catafau et al., 2016).

We detected CF retraction, but not ML shrinkage (Figures 2A,B), as an early architectural change alongside the developmental behavioral deficits in young 4 week old SCA1 mice, perhaps as a direct consequence of disrupted RORalpha signaling (Chen et al., 2013). Interestingly, in 3 week old 30Q (D776) mice (where fewer CAG repeats are overexpressed but a point mutation enhances nuclear translocation of ATXN1) CFs are also retracted (Ebner et al., 2013) and these mice exhibit ataxia 3 weeks later (Duvick et al., 2010). CF retraction is likely to be a critical early change in 4 week old ataxic SCA1 mice, especially as prolonged complex spikes are observed at this age (Hourez et al., 2011), and could arise following the early loss of excitatory amino acid transporter EAAT4 in SCA1 mice (Lin et al., 2000) leading to glutamate spillover and altered mGluR1 signaling (Kano et al., 1997; Ichise et al., 2000; Serra et al., 2006; Power et al., 2016). Understanding the underlying mechanisms of CF retraction as a driver for behavioral deficits could reveal intervention targets for treating ataxia, particularly as CF retraction is seen in human SCA1 (Kuo et al., 2017).

Notably, CF and ML architecture were intact in pre-symptomatic 6 month old SCA1 mice even after 18 weeks of mutant ATXN1 exposure to the adult mouse (Figure 2B). Instead, we observed a specific, but small, decrease in PN soma area in these mice (Figure 4B, and also in SCA1 mice, Figure 4A) that could be a response to mutant ATXN1 activity in the PN nucleus and perhaps also Golgi fragmentation in the soma (Skinner et al., 2002; Emamian et al., 2003). Soma shrinkage could represent the start of the late stage PN loss seen in SCA1 (Clark et al., 1997) and also SCA2 (Huynh et al., 2003). This soma phenotype may also reflect a subtly distinct pattern of mutant ATXN1 gene expression in the adult cerebellum and might usefully benefit from a transcriptome approach (Ingram et al., 2016).

In summary, in this mouse model rapid progression of behavioral SCA1 during development occurs from as early as 4 weeks, starting with CF retraction and later ML shrinkage. Delaying mutant ATXN1 expression until after development prevents behavioral SCA1 and architectural abnormalities for up to 6 months in these mice but may also subtly change the nature of disease progression, starting instead with a modest reduction of PN soma size. Our results highlight the severity of disrupted ATXN1 mediated gene expression in the developing vs. adult cerebellum and emphasize SCA1 in this mouse model as both a developmental and degenerative disorder.

## Author Contributions

MFI and EMP have equal contributions. MFI and EMP: substantial contributions to the conception or design of the work; acquisition, analysis, or interpretation of data for the work; drafting the work or revising it critically for important intellectual content; and final approval of the version to be published. KP: substantial contributions to the conception or design of the work; analysis of data for the work; drafting the work or revising it critically for important intellectual content; and final approval of the version to be published. RME: substantial contributions to the conception or design of the work; analysis, or interpretation of data for the work; drafting the work or revising it critically for important intellectual content; and final approval of the version to be published. All authors to be accountable for all aspects of the work in ensuring that questions related to the accuracy or integrity of any part of the work are appropriately investigated and resolved.

## Conflict of Interest Statement

The authors declare that the research was conducted in the absence of any commercial or financial relationships that could be construed as a potential conflict of interest.
